# A rare and important case of *Staphylococcus haemolyticus*‐associated ventricular atrial shunt nephritis

**DOI:** 10.1002/ccr3.1251

**Published:** 2017-10-30

**Authors:** Kyle Suen, Ardavan Mashhadian, Ian Figarsky, Jeff Payumo, Antonio Liu

**Affiliations:** ^1^ California Hospital Medical Center 1401 S Grand Ave Los Angeles California 90015 USA

**Keywords:** Glomerular disease, nephritis, ventricular atrial shunt, ventriculoperitoneal shunt

## Abstract

Shunt nephritis is a rare and relatively new diagnosis involving glomerular kidney damage following ventriculoperitoneal and ventriculoatrial shunt placement. Our case report summarizes the presentation, diagnostic workup, and management of a patient with shunt nephritis. We also review and discuss the current literature on the topic.

## Introduction

Shunt nephritis is a rare disease of the kidneys caused by bacterial antigen and human antibody immune‐complex deposition in the glomerulus following the placement of a ventriculoperitoneal (VP) or ventricular atrial (VA) shunt. Shunt nephritis is seen more commonly in patients with VA shunts compared to those with VP shunts, and while implantation of VA shunts has decreased as the use of VP shunts increased, there are still VA shunts implanted as a result of problems associated with VP shunts, such as infection or malfunction. Staphylococcus epidermidis, a coagulase‐negative staphylococcus (CoNS), is the most common pathogen in shunt nephritis. Treatment of the shunt nephritis has historically been a combination of three things: 1 prompt administration of antibiotics to eliminate the bacterial infection 2, removal of the infected shunt, and 3, replacement of the infected shunt. Treatment can lead to reversal of the acute kidney injury. In this paper, we will discuss a patient with a confusing presentation of shunt nephritis, as well as briefly review the literature on the topic.

## Case Report

A 43‐year‐old housewife was brought into the emergency department early January by her family members after a witnessed seizure. She was stabilized while a CBC and CMP were ordered. Initial values showed a normocytic anemia, 8.6 million/μL in comparison with a 2014 value of 11.3 million/μL, as well as a severe elevation in BUN and creatinine, 32 mg/dL and 3.9 mg/dL, respectively, and an eGFR of 12 mL/min. Additional laboratory results showed leukocytosis, with a white blood cell (WBC) count of 17.9 thousand/μL. Initial urinalysis was positive for protein (300 mg/dL), red blood cells (1960 RBC/HPF), and white blood cells (88 WBC/HPF). It was revealed that she was being followed up as an outpatient for 3 months for gross hematuria. She was admitted to the hospital for further workup and evaluation of both the seizure and hematuria.

Further investigation revealed an extensive past medical history, briefly summarized in this paragraph. She had previously been diagnosed with hydrocephalus caused by neurocysticercosis 2 years prior. Management consisted of a therapeutic VP shunt placement. She later returned to seek care for recurrent headaches, and was diagnosed with acute obstructive hydrocephalus secondary to shunt malfunction and underwent a shunt revision, which was successful. However, she returned again with recurrent headache. It was determined that her shunt was infected, a complication of the prior revision surgery, and so she underwent a shunt replacement and conversion into a VA shunt.

Upon the current admission to the hospital, an extensive workup for her seizure and hematuria was carried out. The workup of her seizure included a head CT, which was negative for any masses, lesions, or hydrocephalus caused by her existing VA shunt. A cystoscopy came back negative for any lesions or masses in the bladder or lower GU tract, ruling out nonrenal causes of hematuria. Nonglomerular causes of hematuria, such as renal stones, ureteral stones, renal masses, were also ruled out using a CT of the abdomen and pelvis. An abdominal ultrasound showed increased echogenicity of the kidneys, suggestive of parenchymal disease. Pyelonephritis was considered, but a urine culture was negative. Furthermore, the patient did not present with signs and symptoms consistent with pyelonephritis; she denied flank pain, was afebrile on admission, and her initial elevation in WBCs lacked a left shift and trended to normal ranges after her seizure.

Throughout the course of the hospital stay, the patient presented with a variety of symptoms and signs. Her blood pressure was elevated during her stay in the hospital, reaching as high as the 180s/110s mmHg range. She developed anasarca, a grouped maculopapular rash on her hands and feet, nausea and vomiting, and symptomatic anemia that required blood transfusions. Further serum studies showed hyperlipidemia via elevated triglycerides (183 mg/dL), hypoalbuminemia (2.2 g/dL) and an elevated erythrocyte sedimentation rate and C‐reactive protein (ESR and CRP).

Given her history of shunt placement, with subsequent revision and conversion, the diagnosis of shunt nephritis was considered. Blood cultures were drawn, and she was placed on vancomycin and piperacillin/tazobactam empirically. The blood cultures grew *Staphylococcus haemolyticus*, a CoNS, and antibiotic therapy was optimized with the addition of rifampin. A urine sediment was carried out as well, which showed dysmorphic RBCs.

A lumbar puncture was performed, which came back negative for bacterial antigens, WBCs, elevated protein, or decreased glucose. Negative titers for Anti‐dsDNA, Anti‐GBM, and ANA ruled out other causes for glomerular hematuria such as Systemic lupus erythematosus (SLE) or Goodpasture Disease, and her serology for HCV, although suggestive of a latent infection, were negative for active infection. Her complement levels C3 and C4 were found to be low, and she also tested positive for cryoglobulins, which put both shunt nephritis and cryoglobulinemia in the differential diagnosis. However, both diagnoses warranted a renal biopsy, which was carried out.

**Figure 1:**
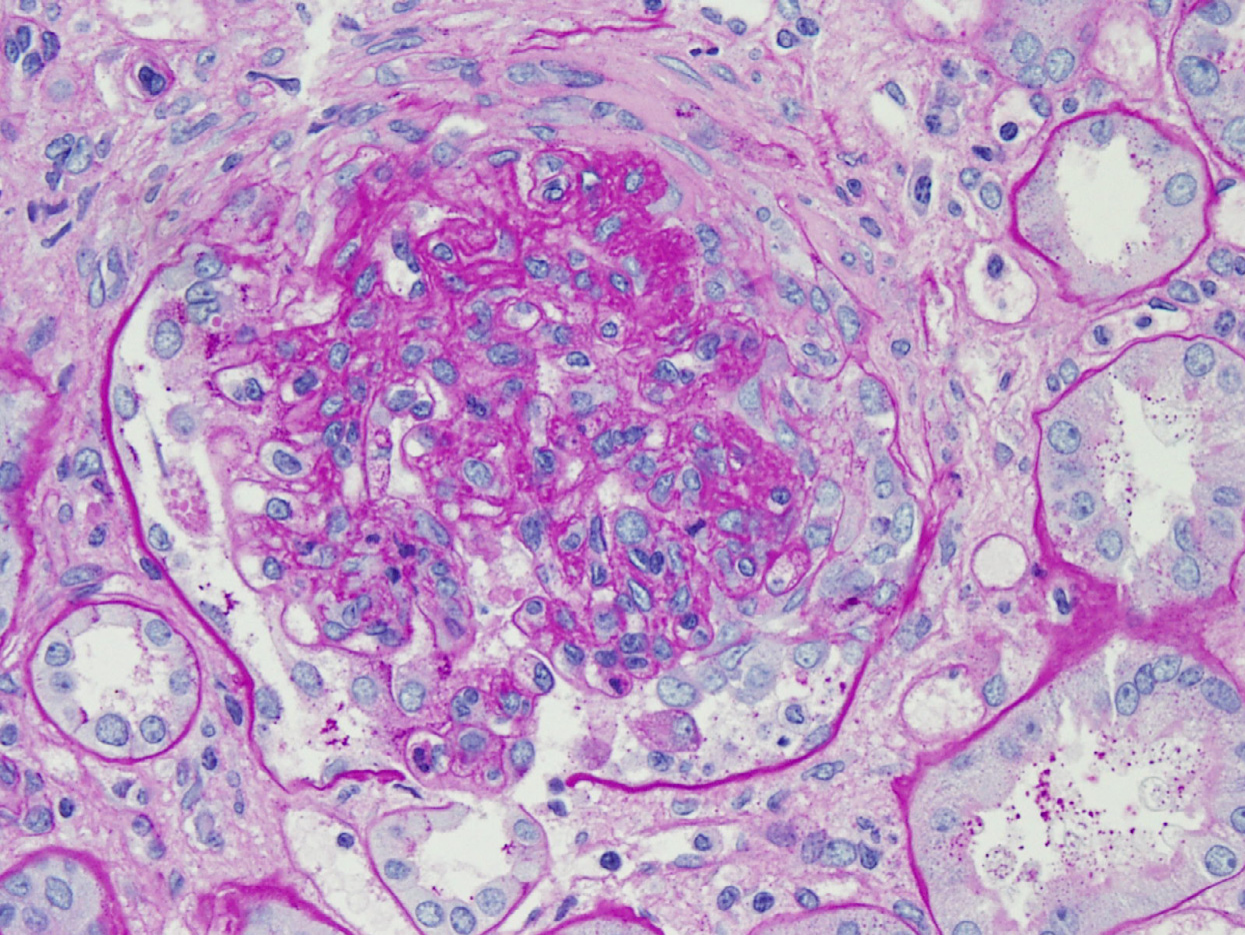
Light microscopy. Glomerulus shows mild to moderate hypercellularity, including mesangial and endocapillary hypercellularity, and a segmental fibrocellular crescent. Some of the hypercellularity is composed of leukocytes; more mononuclear cells than neutrophils.

**Figure 2:**
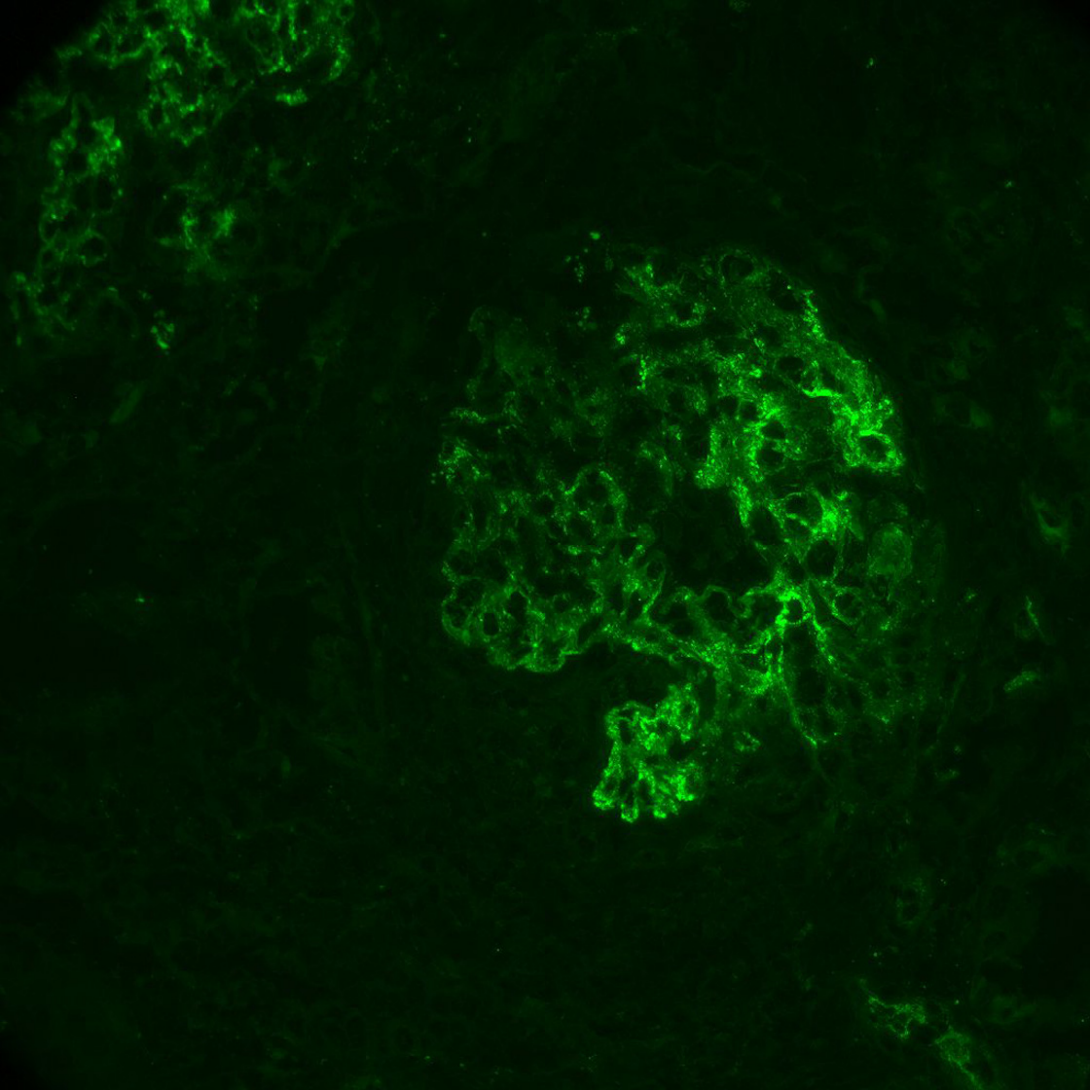
Immunofluorescence. By immunofluorescence, the glomeruli that are not globally sclerotic show fairly diffuse, granular staining in the mesangium with segmental capillary wall extension for IgG (1+), IgM (2+), C1q (2+), C3 (2+) and kappa and lambda (both 1+). There is trace, diffuse, nonspecific pseudolinear glomerular capillary wall staining for albumin. Two glomeruli show blotchy peripheral staining for fibrin, consistent with crescents. There is focal, confluent granular tubular basement membrane staining for IgM (trace to 1+) and C1q (1+). Staining for IgA is negative except staining of tubular casts; these casts also show approximately equivalent staining for kappa and lambda.

**Figure 3:**
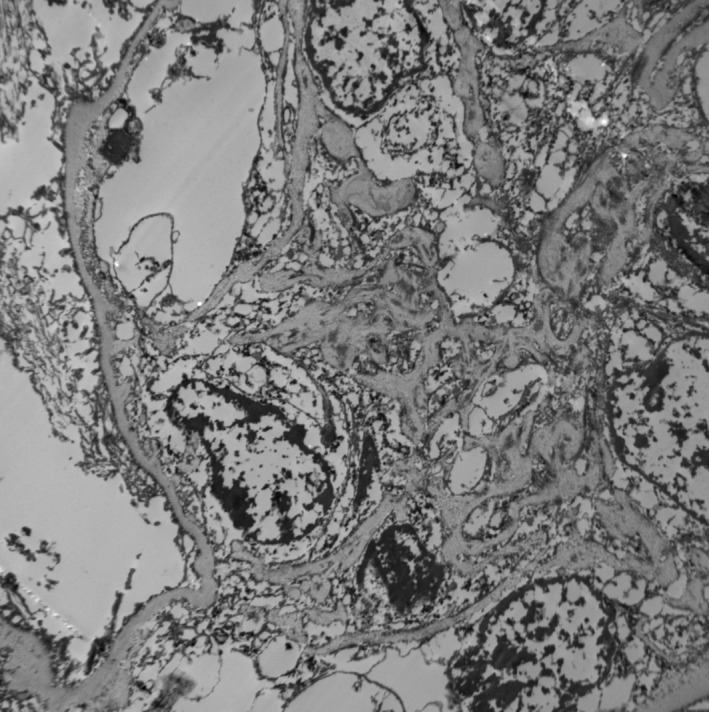
Electron microscopy. Ultrastructurally, there are muiltiple mesangial electron‐dense deposits within expanded mesangial areas. There are segmental, relatively small subendothelial deposits. No subepithelial deposits are seen. There are very focal tubular basement membrane deposits. Podocyte foot processes show extensive but not complete effacement.

The renal biopsy revealed and confirmed the diagnosis of shunt nephritis. Pathology report of the sample showed diffuse proliferative glomerulonephritis with immune‐complex deposits and focal crescents, suggesting shunt nephritis as the most likely diagnosis. The images and comments are shown in Figures 1, 2, 3.

Once the diagnosis was confirmed, the patient was taken to surgery, where the infected VA shunt was replaced with a VP shunt. The tip of the shunt was also sent for culture, which grew *S. haemolyticus*, the same bacteria that was found in her blood cultures.

Postoperatively, the patient's status improved. Her anasarca, maculopapular rash, nausea and vomiting have resolved. Her blood pressures returned to a normal range, 110/63. Her postoperative urinalysis also showed improvement with regard to the proteinuria (100 mg/dL compared to 300 mg/dL on admission) and resolution of the hematuria. Blood cultures postoperatively showed no growth. A few days after surgery, her BUN/Cr was 47 and 7 mg/dL, respectively, and a month after, her BUN was 55 and Cr 3.7 mg/dL. The rest of her postoperative course was unimpressive, and she was discharged a month after surgery when she finished her course of antibiotics. The patient had further follow‐up as an outpatient and her most recent laboratory values showed her BUN had improved to 29 mg/dL, her Cr to 2.40 mg/dL and eGFR of 24 mL/min.

## Discussion

Shunt nephritis is a relatively new and rare diagnosis; there have only been 160 cases documented in the literature as of 2013. The first case of shunt nephritis dates back to 1965, reported by Black et al. 1. He presented two cases of nephrotic syndrome with hematuria. The renal insufficiency seen in both cases was associated with long‐standing CoNS bacteremia. At the University of Nigeria, 212 patients with VP shunts for hydrocephalus were followed over a 13‐year period 2. Of those that developed shunt nephritis, pertinent features included frequent revision of shunts before the onset of nephritis. It was also noted that shunt nephritis is seen more commonly in patients with VA shunts compared to those with VP shunts. The patient's presentation is compared with cases already documented, detailed in Table 1.

**Table 1:** Comparison of clinical features of shunt nephritis in other cases and our patient

Clinical feature	Presence in documented cases	Present in our patient?
Hypertension	39/122 (32%)	Yes
Anemia	108/119 (91%)	Yes
Renal failure	76/142 (54%)	Yes
Hematuria	125/134 (93%)	Yes
Nephrotic syndrome	40/119 (34%)	Yes
Decreased C3	95/107 (89%)	Yes
Staph epidermidis in blood	93/107 (78%)	No^a^
Staph epidermidis in CSF	46/61 (75%)	No
Renal biopsy results		
MPGN	64/107 (60%)	No
DMP	23/107 (21%)	No
EEGN	9/107 (8%)	No
Other	11/107 (10%)	Yes

Data summarized from information found in Haffner et al.

MPGN, membranoproliferative glomerulonephritis; EEGN, endo/extracapillary glomerulonephritis; DMP, diffuse mesangial proliferation.

a Blood cultures grew *Staphylococcus haemolyticus*, another type of CoNS.

Her history of repeated shunt revision and current type of shunt made it more likely that her shunt was the cause of her problems. During her hospital stay, it was determined she had nephrotic syndrome based on her anasarca, persistent proteinuria, and hyperlipidemia. She also presented with symptoms of glomerulonephritis, given her high blood pressures, urine sediment showing dysmorphic RBCs, and her serum complement levels. Her blood cultures indicated a CoNS bacteremia, although it was not the typical S. Epidermidis isolated from patients with shunt nephritis 3.

Our patient lacked signs and symptoms the documented cases presented with. Infections usually manifest itself by the typical signs and symptoms such as a fever or a leukocytosis. The patient was febrile only once at 101.1 throughout her hospital course and had only transient leukocytosis on admission. This isolated leukocytosis lacked a “left shift,” making the etiology of her leukocytosis on admission most likely from the demargination of white blood cells induced postictally and less likely an acute neutrophilic response to infection 4. Prior the literature on shunt nephritis has also shown that many patients who were ultimately diagnosed by renal biopsy did not have the expected signs and symptoms of an infection 5, 6.

It is important to take a moment and discuss the pathogen involved with shunt nephritis. Staphylococcus epidermidis, a coagulase‐negative staphylococcus (CoNS), is the most common pathogen in shunt nephritis. However, the patient's blood cultures and the catheter tip both grew *S. haemolyticus*, another CoNS found as part of normal skin flora. It is common to disregard CoNS‐positive blood culture as contaminant, but when highly suspicious of shunt nephritis, a closer look at these “contaminant” bacteria is necessary for timely diagnosis and initiation of antibiotics. Our patient was started on empiric vancomycin, and piperacillin/tazobactam, and rifampin was added as it had good biofilm penetration that was potentially beneficial, which is a virulence factor characteristic of the CoNS.

The results of CSF analysis of our patient were also unusual. Usually, bacterial shunt infection would show increased protein and WBC, decreased glucose, and the presence of bacteria on culture. The current literature shows 75% of shunt nephritis cases having S. Epidermidis growing in the CSF. Our patient, however, had a result void of WBC's or bacterial antigens as well as a negative culture. One possible explanation is that because the pathogen was different, and that *S. Haemolyticus* behaves differently than S. Epidermidis. However, a more probable explanation is that due to the one‐way valve and the physical properties of the shunt, bacteria and WBCs would be absent from the lumbar puncture depending on the location of the bacteria. Thus, CSF analysis may not support the diagnosis and should not be used to rule out shunt nephritis.

Our patient's renal biopsy is consistent with a progressive, severe form of glomerulonephritis. Once the workup was completed, cryoglobulinemia was also considered given that she developed a rash in the hospital, her hypocomplementemia, and the positive cryoglobulin laboratory assay and its association with HCV infection. However, there have been multiple documented cases of shunt nephritis with positive tests for cryoglobulins, and the patient's renal biopsy lacked intracapillary pseudo thrombi and tubular involvement, making it less likely as the definitive diagnosis 7. The clinical picture and associated labs used in conjunction with the biopsy results made shunt nephritis the most likely diagnosis.

In 2014, Tamber et al. reviewed and discussed the neurosurgical options for dealing with an infected shunt 8. Their results concluded with a moderate degree of certainty that antibiotic treatment with partial (externalization) [of shunt] or complete shunt removal is an option in managing shunt infections. While there is no extensive literature regarding the partial externalization of an infected shunt, the literature has consistently shown that complete removal of the shunt has been associated with resolution of the shunt nephritis 7, 8, 9; our patient supports this recommendation.

Patient recovery has been reported in the literature as following: 54% had complete renal recovery, 18% had persistent urinary abnormalities, 19% went onto ESRD, and in 9% the outcome was death 8. The prognosis of renal function is dependent on how quickly the patient is diagnosed and subsequently, how quickly the infected shunt is removed. In the month following her surgery, our patient's overall condition improved but her kidney function remained limited. In 2014, before her symptoms began, the eGFR was documented as 112 mL/min. On admission, she had an eGFR of 12 mL/min, and her outpatient laboratories 5 months after shunt replacement had increased to 24 mL/min. Unfortunately, her baseline eGFR was unobtainable, as her symptoms of hematuria began 3 months prior to her presentation to the hospital. However, we believe that the shunt nephritis was the cause of the decline in her overall renal function, both the chronic kidney disease, and the acute on chronic disease that caused her to present to the hospital. We suspect that her recovery will likely be that of the 19% going on to ESRD, as the eGFR remained low throughout her stay in the hospital and was still 24 mL/min at her outpatient visit months later.

## Summary and Recommendations

Given that shunt nephritis is a relatively new diagnosis, there have been very few documented cases. Based on the literature and from the patient presentation seen at our hospital, we realize that the typical findings in a patient with shunt nephritis are anything but typical. We recommend clinicians always be aware of and consider shunt nephritis in their differentials for hematuria, as there is no constellation of clinical features that patients will present with.

In our case, the patient's history put shunt nephritis high on the differential. However, the clinical symptoms she presented with and signs seen did not paint a clear picture. She presented with renal symptoms but lacked common findings of a shunt infection, such as fever and leukocytosis. Her diagnostic workup revealed results that did not aid with diagnosis; the rather benign CSF analysis did not show obvious signs of infection. Again, we recommend that a high suspicion based on patient history should guide clinicians to the appropriate workup as signs, symptoms, and lab findings may not accurately reflect the disease process.

Workup for hematuria should be carried out to delineate potential sources of disease, differentiating between nonrenal causes from renal causes, and glomerular causes from nonglomerular causes. A renal biopsy will ultimately be necessary to confirm clinical suspicion. Once the definitive diagnosis is made, management should follow the recommendations made in the literature, including effective antibiotics, removal of the infected hardware, and placement of a new shunt. Again, this is a rare diagnosis that can present in unusual ways atypical of an infection, with diagnostic studies that are also unusual, but it is always important to keep the history of the patient in mind; if there is evidence of acute kidney injury (in our case, acute on chronic kidney injury) in the setting of VA or VP shunts, shunt nephritis should be included in the differential.

## Conflict of Interest

None declared.

## Authorship

KS: was the main contributor to this paper, and did so by drafting the manuscript, analyzing and interpreting data, and revising the article; AM: contributed to this paper by providing critical revision of the article, collecting data, specifically, the microscopy images, and also helping with final approval of the version to be published; IF and JP: contributed by assisting with drafting the manuscript, collecting and analyzing data, and providing the table within the manuscript; AL: contributed to this paper by providing critical revision of the article, getting approval by the institutional board review, collecting data, and helping with final approval of the version to be published.
